# Fundamental constraints to the logic of living systems

**DOI:** 10.1098/rsfs.2024.0010

**Published:** 2024-10-25

**Authors:** Ricard Solé, Christopher P. Kempes, Bernat Corominas-Murtra, Manlio De Domenico, Artemy Kolchinsky, Michael Lachmann, Eric Libby, Serguei Saavedra, Eric Smith, David Wolpert

**Affiliations:** ^1^ ICREA-Complex Systems Lab, Universitat Pompeu Fabra, Dr Aiguader 88, Barcelona 08003, Spain; ^2^ Institut de Biologia Evolutiva, CSIC-UPF, Pg Maritim de la Barceloneta 37, Barcelona 08003, Spain; ^3^ European Centre for Living Technology, Sestiere Dorsoduro, 3911, Venezia VE 30123, Italy; ^4^ Santa Fe Institute, 1399 Hyde Park Road, Santa Fe, NM 87501, USA; ^5^ Institute of Biology, University of Graz, Graz 8010, Austria; ^6^ Complex Multilayer Networks Lab, Department of Physics and Astronomy ‘Galileo Galilei’, University of Padua, Via Marzolo 8, Padova 35131, Italy; ^7^ Padua Center for Network Medicine, University of Padua, Via Marzolo 8, Padova 35131, Italy; ^8^ Universal Biology Institute, Graduate School of Science, The University of Tokyo, 7-3-1 Hongo, Bunkyo-ku, Tokyo 113-0033, Japan; ^9^ Department of Mathematics and Mathematical Statistics, Umeå University, Umeå 90187, Sweden; ^10^ Department of Civil and Environmental Engineering, Massachusetts Institute of Technology, Cambridge, MA, USA; ^11^ Department of Biology, Georgia Institute of Technology, Atlanta, GA 30332, USA; ^12^ Earth-Life Science Institute, Tokyo Institute of Technology, Tokyo 152-8550, Japan

**Keywords:** contingency, convergence, constraints, information, evolution, thermodynamics

## Abstract

It has been argued that the historical nature of evolution makes it a highly path-dependent process. Under this view, the outcome of evolutionary dynamics could have resulted in organisms with different forms and functions. At the same time, there is ample evidence that convergence and constraints strongly limit the domain of the potential design principles that evolution can achieve. Are these limitations relevant in shaping the fabric of the possible? Here, we argue that fundamental constraints are associated with the logic of living matter. We illustrate this idea by considering the thermodynamic properties of living systems, the linear nature of molecular information, the cellular nature of the building blocks of life, multicellularity and development, the threshold nature of computations in cognitive systems and the discrete nature of the architecture of ecosystems. In all these examples, we present available evidence and suggest potential avenues towards a well-defined theoretical formulation.

## 1. Introduction

Imagine that a space probe lands on a distant planet. The probe has sophisticated instruments that detect life on different scales. These instruments might detect a network structure of chemical reactions in the atmosphere or identify molecular biosignatures consistent with living systems [1–6]. The probe can also scan its surroundings, capturing morphological biosignatures and other instruments might analyse the chemical network of the atmosphere and measure its topological and molecular complexity [2,7–9].

How different would such an alternative biosphere be? How dependent would an alternative life form be on the environmental context? Are there physical or chemical pre-conditions required for life to emerge? These questions can be extended beyond evolutionary biology [10] and astrobiology [11] and affect our potential to design (using synthetic biology and bioengineering) novel life forms [12–15]. More generally, we can capture the essence of the previous questions by asking whether we can predict what kind of (possible) living forms of organization exist beyond what we know from our biosphere (the actual).

One established view sees the evolution of life as highly path-dependent [16–18]. In the words of Jacques Monod [16, pp. 42–43],

*…the biosphere does not contain a predictable class of objects or events but constitutes a particular occurrence, compatible with first principles but not deducible from these principles, and therefore essentially unpredictable.*

François Jacob, in turn, discussing tinkering in evolution and the problems associated with levels of complexity, concludes that [19]

*There are always some constraints imposed by stability and thermodynamics. But as complexity increases, additional constraints appear […]. Consequently, there cannot be any general law of evolution*.

Here, Jacob acknowledges the limitations of predicting the properties at one scale based on the properties of the components at lower scales, which is nowadays understood using the concept of *emergent properties* [20,21].

Gould [17] developed the most famous (and controversial) approach to these questions. He argued that because of its historical nature, ‘re-running the tape of evolution’ would lead to entirely different outcomes. Much of the argument was based on the Cambrian Explosion event, which took place 550 Ma ago^1^ and involved the rise of all animal body plans [22,23]. Gould’s work triggered a renewed interest in the Cambrian event and, more generally, in the problem of evolutionary contingency.

The contingency scenario depicted by Gould has been reanalysed and put in a more general context [24,25]. However, the essential message is still relevant within evolutionary dynamics and astrobiology studies. The evidence for convergent evolution has challenged the idea that an alternative biosphere would look alien to ours [10,26,27]. In contrast to Monod and Gould’s views, Conway Morris points out that

*organisms are under constant scrutiny of natural selection and are also subject to the constraints of the physical and chemical factors that severely limit the action of all inhabitants of the biosphere. Put simply, convergence shows that in the real world, not all things are possible*.

The presence of constraints, particularly in the evolution of developmental programmes, was emphasized in the pioneering work of Pere Alberch, who suggested the concept of ‘The logic of monsters’: even when dealing with theratologies, whose phenotypic traits have no selective value, we can organize the diversity of forms under a logic taxonomy, suggesting that organismal complexity is strongly limited [28]:

…*monsters are a good system to study the internal properties of generative rules. They represent forms which lack adaptative function while preserving structural order. There is an internal logic to the genesis and transformation of morphologies and in that logic we may learn about the constraints on the normal*.

In this *structuralist* (or internalist) perspective, gene expression can only be seen as a necessary condition for morphogenetic dynamics, but it is insufficient. As a result of feedback between gene expression patterns and cell–cell interactions, very little of either the structure or the variation in developmental paths is explained by linear mappings from gene labels.^2^

The notion of fundamental constraints is also present in a geometric concept proposed by David Raup: the morphospace, i.e. a multi-dimensional space representing different morphological or structural characteristics of a given class of entities (from cells to networks) [30]. An important lesson from the distribution of living entities across the morphospace is that some parts are densely occupied while others are voids, associated with unobserved possibilities. This uneven occupation is strongly related to the role played by constraints and the presence of convergent solutions. This concept has been extended across disciplines, from network topology to language [31,32], and also applies to ecological systems [28,33]. Like morphospaces, species-interaction networks partition the environmental parameter space into a discrete set of possible biotic configurations, where some partitions can be larger than others [34,35]. This robustness (compatibility with a larger set of environmental conditions) can be a target of evolution, as argued by Waddington and others [36–38].

Finally, there is an argument by Stuart Kauffman and collaborators concerning the intrinsic unpredictability of evolutionary dynamics due to the ‘non-ergodic’^3^ character of biology [39]. In their own words [40,41]:

*The chemical and physical properties of the different complex molecules are different, and in biology, the functional properties of these tens of thousands of different molecules in cells are also different. The universe is not ergodic because it will not make all the possible different complex molecules on timescales very much longer than the lifetime of the universe. It is true that most complex things will never ‘get to exist’*.

The authors use ‘non-ergodic’ here in the sense that not every part of the configuration space can be explored. In a nutshell, the size of sequence space Ω associated with a biological polymer of length L built from a molecular alphabet Σ of size |Σ| would be


$$\Omega ∼|\Sigma {|}^{L}.$$

Thus, the space of possible proteins with a length of 1000 amino acids is $20^{1000}$, a space so large that it could never be explored in our universe [42]. The space of possible molecular configurations of molecules within an organism is yet astronomically larger. How can we then talk about universal life features in this scenario?

Nonetheless, there are several major reasons to expect convergence. First, all evolutionary trajectories occur under certain generic selective constraints, such as the laws of mathematics, physics and chemistry, and these should lead to some universal features [43,44]. Second, many of these spaces are not explored randomly. For example, analyses of the nature of the genotype spaces show that network structures are far from uniform [45]. This is particularly relevant when dealing with the emergence of molecular functions, where the genotype space is highly redundant [46], meaning that a huge number of genotypes are consistent with the same phenotype [47]. The percolating nature of these genotype spaces strongly favours the potential for success in evolutionary search [48]. Quoting Susanna Manrubia, these properties *further ensure that different functions may await just a few mutations apart* [46,49,50]. Moreover, some intrinsic properties of chemical and physical nature can deeply constrain the possible repertoires of molecular structures, as exemplified by the fact that only a small fraction of protein folds are realizable even looking at the full sequence diversity [51–54]. Finally, evolutionary trajectories happen within a certain system: Darwinian evolution will act on populations of entities. Entities within the system emerge within assembly spaces [2,55,56]. They are constructed from components over evolutionary timescales. These shape evolution and shift the way one should think about sequence search conditioned on the past, where the logic of assembly spaces may lead to certain types of universal convergence [56].

Here, we discuss some of the fundamental constraints that limit the space of evolutionary outcomes. We focus on areas that are most well-studied and most likely to be universal, involving several case studies that reveal deep constraints associated with the *logic* of the organization of living systems. Specifically, we start with some core thermodynamic constraints and then discuss the logic underlying molecular information carriers, cellular reproduction, multicellularity (MC), cognitive architectures and ecosystem organization. Finally, we consider the concept of phase transitions as a paradigm for the emergence of living complexity. A recurrent theme in the following sections concerns predictions about biological complexity made before empirical evidence came in. In our view, such predictions strongly hint at some kind of universality. Such universality fits within the broader goal of finding general theories of life that transcend the specifics of life on Earth [44,57–71].

## 2. Living systems as thermodynamic engines

Every organism on Earth operates as a thermodynamic engine because it acquires free energy from the environment and uses it to drive essential biological functions. The ubiquitous nature of thermodynamic constraints suggests that a certain kind of *thermodynamic logic* is a universal feature of living systems. In this context, thermodynamics allows us to address the problem of the bounds to the efficiency of living, information-processing agents [72–76].

Any form of life is expected to be an embodied and differentiated structure that performs healing, self-repair and error correction. Such processes reduce the system’s entropy by mapping a large set of ‘incorrect’ (damaged) states to a much smaller set of ‘correct’ (viable) states. Entropy reduction also comes by growth: the synthesis of organized biological machinery from simpler, disconnected components—for example, as done by the ribosome during the synthesis of proteins—involves a large reduction in entropy [77].

The second law of thermodynamics states that a physical process must generate an overall increase $\Delta S_{\text{total}}$ of the entropy of a system ($\Delta S_{\text{system}}$) and its environment ($\Delta S_{\text{env}}$):


(2.1) $$\begin{array}{ccc} & \Delta S_{\text{total}}=\Delta S_{\text{system}}+\Delta S_{\text{env}}>0. & \end{array}$$

Therefore, living systems can only perform processes that reduce entropy internally if, at the same time, they produce an even greater increase of entropy in the environment. Thus, the universal thermodynamic logic of life is that low-entropy input (‘resource’) is turned into high-entropy output (‘waste’), which can be expressed as^4^


(2.2) $$\begin{array}{r} \Delta S_{system}<0,\;\Delta S_{env}>0,\;|\Delta S_{\text{env}}|>|\Delta S_{\text{system}}| \end{array}.$$

This suggests that all life forms face the problem of maintaining a low internal entropy in a race against the second law. This thermodynamic perspective on biology is closely associated with Schrödinger’s well-known 1944 book *What is life?* [79] and the much less well-known articulation by Boltzmann in 1886 [80].

We may add a few important details and questions to the above-mentioned general picture. First, for a system coupled to a thermal reservoir at temperature T, such as an atmosphere or an ocean, the entropy production in the environment is related to the amount of heat released Q,


(2.3) $$\begin{array}{ccc} & \Delta S_{\mathrm{env}}=Q/(k_{B}T), & \end{array}$$

where $k_{B}$ is Boltzmann’s constant. In fact, for almost all life on Earth, thermodynamic driving is accomplished by acquiring energy from the environment and releasing it as heat. Important examples of heat generation include the absorption of visible photons and radiation of lower-frequency infrared, the breakdown of high-energy biotically produced molecules (within trophic ecosystems) and the exchange of electrons between environmentally provided donors and acceptors [81]. Although, in principle, entropy production can also occur due to exchanges of other quantities besides energy [82,83], heat flow is a fundamental and ubiquitous energy-exchange channel, suggesting it should have a primary role in the thermodynamics of life on other planets.

Biological heat generation recapitulates the pattern observed at the planetary scale. Late-stage planets are essentially closed to exchanges of matter with their environments but open to energy exchange via incoming solar flux and outgoing thermal radiation [84,85]. Notably, the other source of thermodynamic driving available to chemotrophic life—the gradual oxidation of mantle minerals—needs to be coupled to hydrogen escape to maintain more oxidizing surface conditions than the interior in an era without photosynthetic production of oxidants [86]. However, on a planet with oxygenic photosynthesis, even that process can be maintained while conserving matter, as long as the burial of organic carbon can compensate for the liberation of oxygen—a large-scale geological rearrangement driven by sunlight but mediated by life. To summarize, it is very likely that heat generation serves as a primary thermodynamic driving force for life anywhere. To the extent that life does not completely replace geological processes with novel ones [87], but rather partially conserves geologically facile processes [86], the sharing of major thermodynamic properties by the two becomes even more expected.

A second important point is that the second law is obeyed not only globally—that is, at the level of the entire organisms—but also locally at the level of each individual reaction. For this reason, each entropy-reducing reaction must be locally coupled to a free-energy source.

In biology, a collection of energy intermediates drives many biotically essential transitions that otherwise would not occur spontaneously [88,89]. These intermediates include, first and foremost, the phosphate-bearing cofactors (ATP and the other nucleoside triphosphates, and others [90]) that can drive dehydrating reactions by phosphoryl group transfers, a variety of electron-transfer cofactors (such as NAD, NADP and a variety of others [91]) and membranes that act as capacitors for the exchange of protons. Energy intermediates remove the need for internal processes to be in direct contact with environmental sources of free energy, thus achieving a kind of thermodynamic autonomy [92]. In addition, the fact that many core reactions coupled to these intermediates can be run bidirectionally [93,94] allows life to store energy, e.g. in molecules like glycogen, thereby buffering against stochastic and deterministic (e.g. day/night) environmental fluctuations and achieving further thermodynamic autonomy [92].

**Figure 1.**
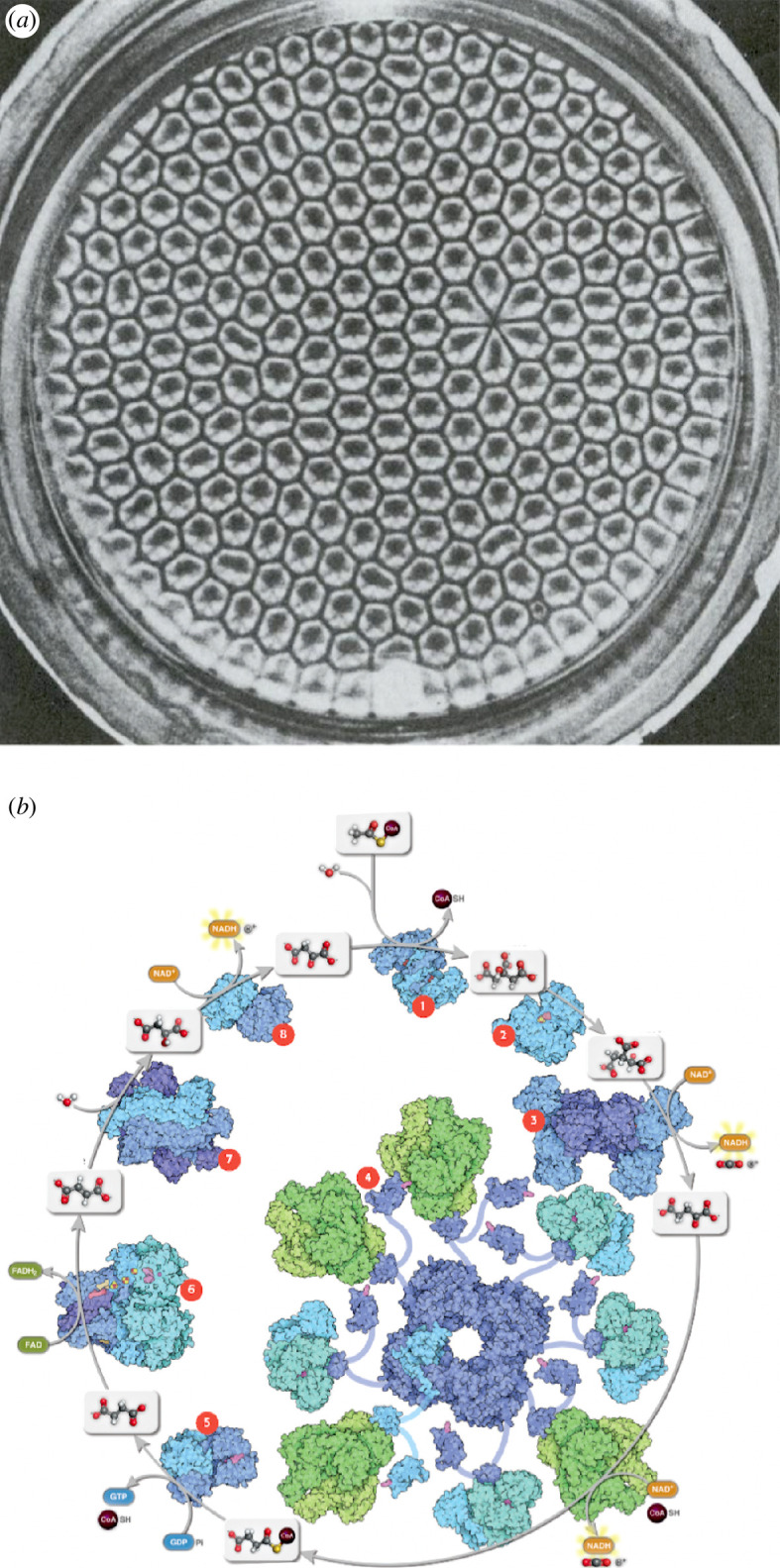
Cyclic structures characterize dissipative systems that reach non-equilibrium steady states due to external driving. In an abiotic system like a Bénard cell (*a*), a temperature gradient leads to the formation of cells that transport heat by cyclic convection (image adapted from Koschmieder & Pallas [97]). In living systems, chemical energy drives metabolic cycles such as the citric acid cycle (*b*), which plays a crucial role in energy production and biosynthesis. Such metabolic cycles consume resource molecules and synthesize energetic intermediates and building blocks while releasing heat. In the figure, both the intermediate metabolites of the cycle and the enzymes are indicated (image from David Goodsell).

As a third point, it is interesting to consider what can be added to the picture using results from non-equilibrium thermodynamics, such as Onsager’s principle of detailed balance [95]. One important insight made by Morowitz [96] is that any non-equilibrium chemical system in steady state must exhibit cycles, as illustrated in figure 1. That is, it must exhibit sequences of transformations that leave the system’s state invariant while exchanging energy and/or matter with the environment. Today we recognize metabolic cycles, such as the citric acid cycle [98,99], as some of the most fundamental and universal organizing principles of metabolism. Morowitz’s insight was that such metabolic cycles must be present in any living system (see figure 1*b*).

To illustrate this concept formally, we may consider a simplified model involving N metabolites, denoted by the vector $𝐂=(C_{1},C_{2},\ldots ,C_{N})$. Suppose that the concentration of each metabolite can be influenced by the reactions converting it from and to other metabolites,


(2.4) $$\begin{array}{r} \frac{dC_{i}}{dt}=\sum_{j:j\neq i}J_{ij}, \end{array}$$

where $J_{ij}=-J_{ji}$ is the net flux of the reaction that converts metabolite j to i. In a steady state, all concentrations are constant, so $dC_{i}/dt=0$ for all i. The key point is that outside of the trivial (equilibrium) steady state where all fluxes vanish ($J_{ji}=0$), steady-state fluxes must necessarily exhibit one or more cycles [96,100]. An example is provided by a cycle such as 1→2→3→1 that corresponds to positive fluxes $J_{21}=J_{32}=J_{13}=1$. This cycle involves the creation and destruction of metabolites 1,2,3, so its net effect leaves the concentrations invariant.

While living systems must meet the constraints of energy, it should be noted that more proximal constraints from kinetics, from limits of chemical mechanisms or from robust paths for construction and degradation may impose tighter constraints that obscure ultimate roles from thermodynamics. The complexity of identifying the relative importance of ultimate versus proximal constraints is a chief source of difficulty in interpreting based on idealized reversible assembly [101]. However, how key a constraint energy is to living systems can be tested by assessing how close organisms have come to the possibility frontier. Efforts to assess the conversion efficiency in heterotrophs from food to new biomass have been made [102], including a very early effort by Morowitz [103] to tie these explicitly to entropy. More recent work has sought to include the more subtle entropies of sequence information [77] within estimates of efficiency for biological assembly. Other arguments related to metabolic scaling indicate that energy is a key consideration in the evolutionary optimization of organisms [43,44,104,105].

A final note to properly frame the extent of thermodynamic constraints is needed. Many abiotic systems exhibit some of the properties mentioned above. For example, hurricanes, Bénard cells (figure 1*a*) and other naturally occurring ‘dissipative structures’ [106] also couple local entropy reduction to external entropy production and also exhibit ongoing cycles, yet they are not alive. However, apart from biosynthetic networks in organisms and a few engineered synthetic chemical systems, there are no known dissipative structures whose cycles drive the chemical assembly of complex molecules rather than the mechanical formation of physical structures (such as vortices). Another difference is that abiotic dissipative structures depend entirely on the presence of appropriate boundary conditions for their existence. This is unlike living systems, which construct organismal boundaries and maintain internal energy stores in order to attain a degree of thermodynamic autonomy from immediate environmental conditions.

## 3. Linear information carriers

Information plays a central role in living systems beyond energy and matter and the thermodynamic considerations described in the previous section. Previous work has suggested that some of the most universal characteristics of life are related to its informational, algorithmic and computational properties [44,62,70,107].

Here, we consider one aspect of this story, motivated by the fact that all lifeforms seem to require physical information carriers that provide the means by which phenotype properties can be transmitted across generations or time. Two properties of information carriers are important. First, they can reliably code for a large number of phenotypic states. Second, that information can be replicated. Without the emergence of a shared information carrier, any evolved feature also has to evolve a method of transmission of that feature—almost like a new origin of life. What are the fundamental constraints associated with biological information carriers?

Of course, the best-known information carrier in biology is the DNA molecule. Two remarkable insights into the nature of genetic information were advanced before molecular biology and the unveiling of DNA structure in 1953. The first has to be found in the writings of Nikolai Koltsov, who, as early as 1927 concluded that the basis of heritability at the molecular and cellular level had to be found in some class of giant, double-stranded molecules able to self-replicate in a semiconservative way [108,109]. Koltsov’s conjecture was that the genetic material replication should be explained in terms of linear copolymers, with each strand to be used as a template [110]. Seventeen years later, another suggestion was found in Schrödinger’s book *What is life?*, where he suggested that the information-coding molecule (the gene, pp. 60–61 in [79]) should, on the one hand, be a regular structure (like a ‘crystal’) while, on the other, allow for an intrinsic ‘disorder’ compatible with ‘atoms playing an individual role’.^5^

**Figure 2.**
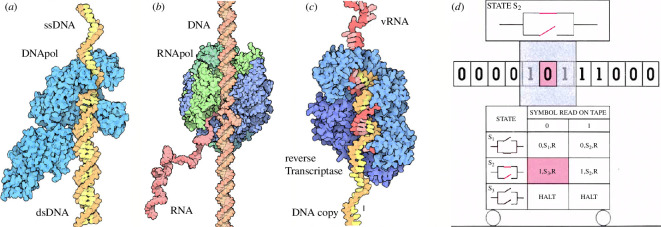
Linear polymers, information and computation. Several molecular events involve information storage and processing, such as DNA replication (*a*) or transcription (*b*) or the RNA → DNA reverse transcription in viruses (*c*). All these examples involve linear polymers that are ‘read’ by special nanomachines (DNA and RNA polymerases or the reverse transcriptase). Here, RNApol is RNA polymerase, DNApol is DNA polymerase, ssDNA is single-stranded DNA, dsDNA is double-stranded DNA and vRNA is viral RNA. The classical model of computation defined by Turing (*d*) involves a machine with internal states that scans a linear string of symbols (here made of zeros and ones) and changes its internal states as computation proceeds. Images (*a*) and (*b*) are adapted from David Goodsell. Image (*c*) adapted from Hopcroft [117].

Koltsov’s and Schrödinger’s visions, based on reflections turned out to be strikingly accurate once Watson, Crick and Franklin uncovered the structure of DNA [115,116]. Their work revealed a right-handed helix composed of two antiparallel strands twisted around each other, forming a helical backbone of sugar–phosphate groups with nitrogenous bases paired in the core. As predicted by Koltsov, the discovery elucidated the mechanism of heredity based on semiconservative replication and laid the foundation for understanding how genetic information is stored and transmitted in living organisms. Here, DNA molecules are copied thanks to DNA polymerases, which can ‘read’ single-stranded DNA chains^6^ that act as the ‘tape’. This also includes a proofreading mechanism: the DNA polymerase can detect errors in the base pairing and remove the mismatched nucleotides. Moreover, linear tapes and their reading machines are also at work at the levels of transcription (RNA synthesis from DNA; figure 2*a*) and translation (protein synthesis from RNA; figure 2*b*). These systems can be classified as biocomputing machines within a hierarchy [118,119].

Beyond DNA, life, in general, uses three types of polymers: polynucleotides, polypeptides and polysaccharides [120]. Because of their particular chemical features, information and functional (enzymatic and structural) machinery are associated with the first two, while the third group is responsible for energy storage and recognition. The mapping between polymer class and its role is tightly related to their folding, assembly and complementarity properties. Folding allows for the formation of low-entropy states that become stable, thanks to self-interactions, whereas in polynucleotides, monomers do not engage in attractive interactions and display energy degeneracy, i.e. each sequence is energetically about equal. Sugar-based polymers, on the other hand, are branched structures made of the same class of monomers.

The widespread use of long, linear polymers for biological information processing is striking. Is there anything special about one-dimensional linear polymers that leads them to be a universal solution to the information-carrying problem? Are higher-dimensional information carriers possible? Three main arguments support the idea that the linear polymer is the expected option, based on constraints associated with (i) evolvability, (ii) computation and (iii) thermodynamics. Several authors have discussed the first, which concerns the enormous advantages of *some* kinds of linear polymers. As pointed out by Howard Pattee [121] (see also [122–126]):

*There is an enormously larger class of natural structures that have nearly equal probabilities of formation because they are one-dimensional and have nearly equivalent energies. They are linear copolymers, like polynucleotides and polypeptides. Life and evolution depend on this class of copolymer that forms an unbounded sequence space, undetermined by laws. […] This unbounded sequence space is the first component of the freedom from laws necessary for evolution*.

At the same time, not all information-carrying and information-processing systems are one-dimensional in molecular biology. For instance, the folded DNA molecule exhibits a rich three-dimensional spatial organization, much of which has important functional roles in regulating gene expression. At a more abstract level, cells display a tangled web of molecular interactions connecting different (genetic, metabolic and signalling) networks, which are appropriately conceptualized in high-dimensional space. Nonetheless, much of this complex organization emerges from the capabilities enabled by underlying one-dimensional molecules. This view is partly supported by the observation that higher-dimensional forms of information processing (whether in physical space as with chromatin or abstract space of gene regulatory networks) appear to play a smaller role in more primitive lifeforms, such as prokaryotes.

Beyond the specific solutions found in modern cells, let us consider the following question: what would be the expected logic of a minimal information-processing molecule? This is a relevant problem since any early life capable of evolution should have been able to store and propagate information. Given our current knowledge of molecular cell biology, we might be biased while answering the previous question. In modern cells, linear chains made of discrete units from a given alphabet store information and are scanned by another given molecule that can read the message. However, such a picture was already in place before molecular biology, which Alan Turing introduced in 1936.

The so-called Turing machine is a mathematical model of computation, and its definition will sound very familiar. It consists of an infinite tape divided into cells, a read/write head that moves left or right along the tape and has a finite set of states. Each tape cell contains a symbol from a finite alphabet (figure 2*c*). The machine operates based on a set of transition rules: given the current state and symbol under the head, the machine can write a new symbol, move the head left or right and transition to a new state. The Turing machine represents a foundational concept in the theory of computation, defining a simple computational mechanism capable of performing any algorithmic computation—thereby, it is universal and establishes the basis for understanding the limits and possibilities of algorithmic processes. While building such a general framework, Turing’s ideas (perhaps inspired by some of the technology of his time, such as tapes and machines reading them) surprisingly match the molecular computational devices resulting from evolution.

Chemically speaking, some linear polymers seem the simplest and most reliable choices if we need to store bits on a molecular structure. For obvious reasons, a homopolymer (i.e. made of identical monomers) would carry no information. Instead, a heteropolymer, formed by a chain of different kinds of monomers, would provide the substrate of many possible strings of symbols while allowing evolution to occur. Additionally, a molecular system able to scan this string would be strongly constrained by the one-dimensional nature of the heteropolymer. In this context, we could imagine an alternative molecular machinery where a given information substrate is based on some heterogeneous two-dimensional set of monomers and such potential is illustrated by the remarkable advances in DNA engineering as a material [127,128]. Beyond these nanostructure assembly processes,^7^ it has been shown that two-dimensional monolayers can be obtained from mixtures of adenine and uracil [129,130] leading to aperiodic structures, although under contrived physical conditions. In this context, despite the potential information that can be stored in these monolayers and the relevance of surfaces to facilitate polymerization [131], polymers can eventually end up strongly bound to the surface, becoming an evolutionary dead end. Beyond these possibilities, it is not difficult to imagine the challenges imposed by creating, reading and replicating such kind of molecular information in predictable ways.

Our central interest here has been to go beyond the specific to the universal. What can be said of information-processing systems in general? One fundamental constraint is the energy required to perform information-copying operations. Landauer’s bound [132] gives us the minimal energy required to perform an abstract computation and many string writing operations, including copies and transformations of it [133]. Copying a DNA strand or producing a protein from a ribosome can be cast as writing a specific string from a set of unordered letters (e.g. nucleotides or amino acids). Under this framework, it is possible to calculate the minimal energy required to write a string, and amazingly, it has been shown that the energy usage of the ribosome is only one or two orders of magnitude above this bound [77].

This is an interesting case where fundamental physical limits are relevant to a very general process, and they also help us to understand cellular physiology. For example, how should we assess whether the energy flux of an environment is sufficient to support a living system? One extreme possibility is to ask if the available energy is sufficient for string copying and processing operations for a very small amount of stored information. One can easily connect the fundamental bounds of information to metabolism via Landauer’s bound, by calculating the minimal metabolic rate W needed to replicate genetic information given a genome length and desired cellular growth rate [77]. Landauer’s bound states that [132]


(3.1) $$Q\geq k_{B}T\left(S_{I}-S_{F}\right),$$

where Q is the heat released to a bath at temperature T, $k_{B}$ is Boltzmann’s constant, $S_{I}$ and $S_{F}$ are the initial and final system entropy, respectively. This is nothing more than the expression of the second law of thermodynamics, equations (2.1) and (2.3), where $\Delta S_{\text{sys}}=S_{F}-S_{I}$. For the case of writing a specific string from a set of unordered letters, we have that $S_{F}=0$ and


$$S_{I}=\ln\left(n^{L}\right)=L\;\ln\;n,$$

n is the number of unique elements, or letters, in the informational system and L is the length of information (the string length) that is being copied. We can convert this into a metabolic rate by considering how fast the information is copied. Given a time to divide $t_{d}$ in seconds, the minimal metabolic rate of copying is


(3.2) $$W=k_{B}T\frac{L\ln n}{t_{d}}\;\text{J}\;{\text{s}}^{-1}.$$

**Figure 3.**
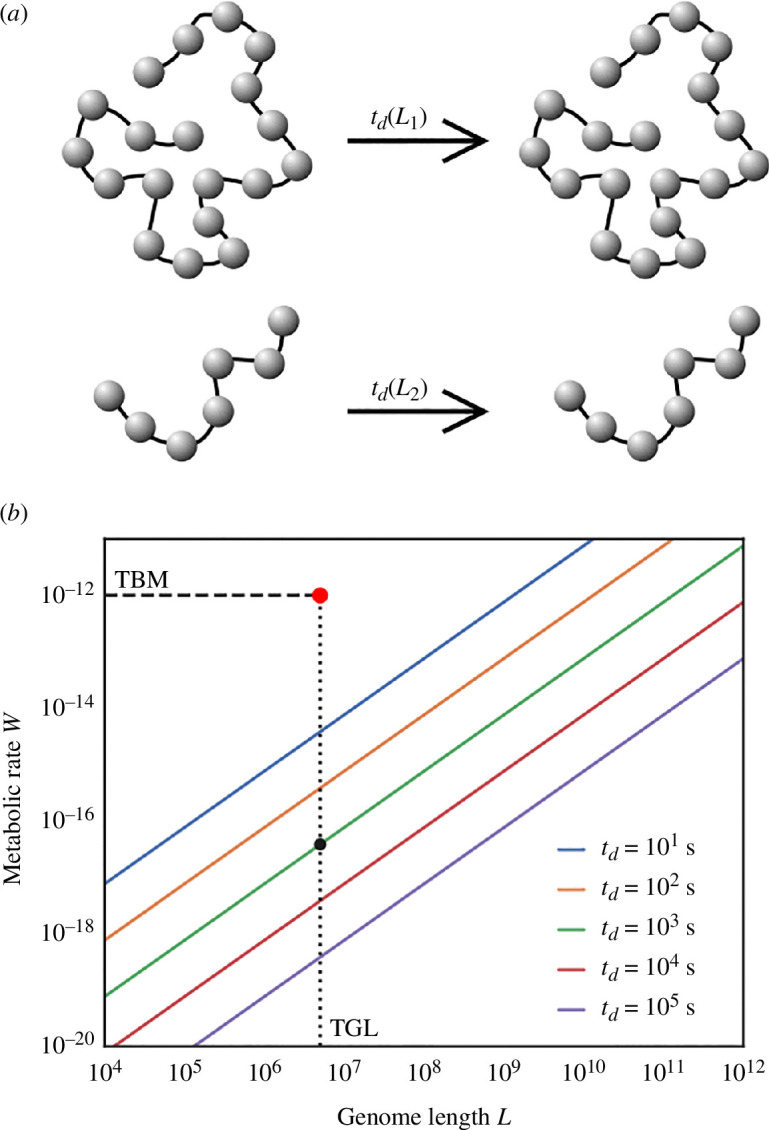
Required metabolic rate for information copying alone, as calculated by equation (3.2). The key parameters are the number of letters in the informational alphabet (n), the length of the genome (L) and the time to copy the information ($t_{d}$). We show the minimal metabolic rate for n=4 at various division times $t_{d}$, from 1 s to $10^{5}$ s. Dashed lines indicate a TBM rate of $W\approx 10^{-12}$ J s^−1^ [134], TGL of $L\approx 5\times 10^{6}$ bases, with typical division time $t_{d}\approx 10^{3}$ s.

We illustrate this result in figure 3 for an alphabet of n=4 elements for various L and $t_{d}$. This is the minimal metabolic rate for copying alone and does not account for other functions of an organism. We reference the typical bacterial metabolism (TBM) and the typical genome length (TGL) of bacteria. For typical division times, the minimal genome copying cost is many orders of magnitude smaller than known metabolic rates. However, as shown in figure 3, the costs increase with larger genomes and shorter division times.

## 4. Cells as minimal units of life

All living entities in our current biosphere can be classified as cellular life forms and virus-like elements [135]. Cell division involves, on the one hand, copying the information contained in the cell to its daughter cells, and on the other, reproduction of its embodied architecture by using this information^8^ of its embodied architecture; each copy must define a compartment and a set of metabolic components necessary to start a new cell cycle. More abstractly, both genomes and their vessels require constructive processes. For the genome, the constructive partitioning and assembly occurring in most genotypes’ life cycles operate over the more basic copying dynamics, as Watson, Crick and Franklin recognized. The term ‘construction dynamics’ has been introduced [136,137] to study specific structural factors, ranging from reproduction to ecology and niche construction [138], that place universal constraints or requirements on the dynamics of evolving populations.

In our biosphere, the copying of genomes ultimately depends on the reproduction life cycle bound of metabolizing cells. In that sense, even if they are not singular or even ‘minimal’ by any unambiguous measure, they are *essential* to the realization of living states on Earth [139–143]. Even the simplest cells exhibit an extraordinary and diverse molecular complexity and common design principles. As far as we know, no alternative building blocks exist in our biosphere. Is the logic of cellular organization an inevitable outcome of the evolution of life? Here, we consider three critical constraints that might impose some fundamental limits to what such a living autonomous agent might be. These include the following three main concepts: (i) the logic of self-replicating machines, (ii) the physicochemical logic of minimal autonomous agents (cells) that become differentiated from their external environment, and (iii) the thermodynamic limitations associated with reliable reproduction.

**Figure 4.**
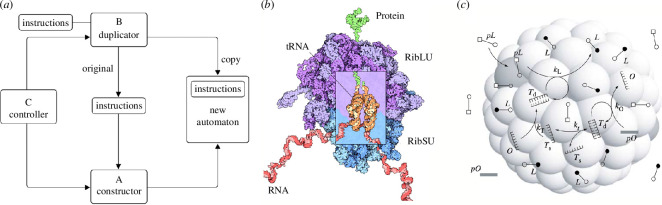
The logic of self-replicating living ‘machines’. Cells reproduce through a complex process that uses DNA as a set of instructions but requires also DNA to be replicated. In von Neumann’s theory (*a*), a formal machine capable of copying itself would require a set of instructions to guide the construction of a new machine under some controlled states such that instructions also get replicated. In biology, one crucial component of the cellular translation machinery is the ribosome (*b*), made of two subunits (RibLU and RibSU) that ‘read’ RNA strings to synthesize proteins, playing the role of the Constructor. In a molecular, embodied implementation of cell reproduction, self-organized interactions between a compartment, metabolism and information must interact. An example of a simple implementation is shown in (*c*) for a synthetic cell involving a compartment coupled to double-stranded polymers and a minimal metabolism (adapted from Munteanu *et al*. [146]). Here, a precursor pL is transformed into lipids (L) that allow membrane growth until some instability triggers division.

The algorithmic basis of self-replication was approached by von Neumann using a very abstract (but also general) view, thus ignoring the exact nature of the physical components and the specific functions carried out by the replicator. Von Neumann understood the importance of information and its relevance in providing the instructions necessary to reproduce the entire system while also copying the instructions themselves.^9^ To some extent, von Neumann’s so-called *Universal Constructor* (UC) was inspired by the steps followed in a factory to build machines in an assembly line, where each component has to be available in space for assembly. Formally, it was defined in terms of a ‘machine’ that is implemented using operations on a lattice. The machine includes the following four primary components: the Constructor, the Instructions, the Duplicator and the Controller (figure 4*a*). The Constructor (A) builds the new machine out of components from the surrounding environment. The Instructions (I) contain information on how A will operate and effectively define an input tape (as in Turing machines). The Duplicator (B) reads the instructions and duplicates them. Finally, the Controller (C) regulates the whole process, which has to unfold in a given sequence. As defined, the tape plays two markedly different roles. First, the information on the tape provides instructions to be interpreted and allows the construction of a machine. On the other hand, the information on the tape is also treated as uninterpreted data, which must be copied and attached to the new machine.

Von Neumann’s insight went a crucial step beyond Schrödinger’s conceptualization of information by showing that a self-replicating agent must contain a sufficient description of itself [147,148]. As happened with our previous case study, the components of von Neumann’s construction mirror those of self-replication found in cellular biology. Although the biological reality is significantly more complex and multifaceted, we find close similarities between the Duplicator and the information storage mechanism in cells (DNA, perhaps RNA in early protocells), the Controller’s role in interpreting and executing instructions resembling cellular control mechanisms, the Constructor’s function in manufacturing new components akin to cellular machinery (as executed by RNA polymerase and the ribosome; see figure 4*b*) and the Instructions reflecting the genetic information directing cellular self-replication. These striking similarities suggest a fundamental logic determining the critical components required for a self-replicating system. Once again, von Neumann’s theoretical insight came years ahead of discovering the relevant structures in molecular biology. No less important, von Neumann’s insight went beyond the problem of self-replication and is deeply ingrained with the problem of open-ended evolution, which has been a central problem within the field of artificial life [149–152] by pointing to the minimal conditions for complexity to be able to grow.

The search for other formal systems able to self-replicate, usually defined on a two-dimensional lattice, has shown simpler examples with a much smaller number of parts than those proposed initially by von Neumann [64,153]. However, a rather crucial problem exists when mapping the original cellular automaton approach to the UC into the real world: all these systems share a high brittleness. Due to the deterministic, spatially dependent nature of the rules required to implement replication, even a slight error (or mutation) typically destroys the whole pattern. Initial conditions must also be fixed in some predetermined way; otherwise, the system will not follow adequate paths towards reliable copying.

How can we solve it? The qualitative theory put in terms of the UC design is agnostic as to how one implements it, and one answer to our question comes from the dominant role played by self-organization of soft matter [154,155] and the requirements to obtain a system displaying agency. Here, chemical and physical constraints play a crucial role. In looking for them, we will also see that alternatives lacking information (a set of instructions) can exist, defining a compartment-metabolism system. More importantly, understanding these phenomena and the potential presence of constraints is essential to understanding the transition from non-living to living matter and the design of artificial cells [142,156–160].

The first constraint for a system to be able to replicate itself is very basic: molecular components need to be available. This is achieved when there is phase separation from the environment [143,161,162] that can concentrate the needed substrates within a given domain. In practical terms, this requires a closed compartment defining an inside and an outside and the subsequent flows of energy and matter that occur across the boundary. Interestingly, a rather limited set of structures are the most plausible candidates. In a water-solvent world, one robust path to creating a compartment that separates the inside from the outside environment is provided by amphiphiles, which spontaneously self-organize in space to form well-defined structures. They are polar molecules with a well-defined hydrophilic head group (attraction to water) and a tail group showing hydrophobicity. Due to this conflicting relation towards water molecules, amphiphiles can self-assemble into compact bilayers that define the system’s boundaries. Along with this polar nature, the shape of one molecule largely decides the curvature of the self-organized assembly. The bending energy associated with a given closed configuration is given by


$$H_{b}=\oint_{S}\frac{\kappa [S]}{2}(C(S)-C_{0}(S){)}^{2}\;dS,$$

where κ[𝐒] is the bending modulus and $C(𝐒)-C_{0}(𝐒)$ is the mean curvature of the vesicle surface at 𝐒. Energy minimization, as defined from the solutions of $\delta H_{b}=0$, provides a wide range of possible shapes, from highly stable (spheres, for example, with only positive curvature) to others displaying metastable states that imply the presence of local negative curvature (required when cell division occurs) [163,164].

**Figure 5.**
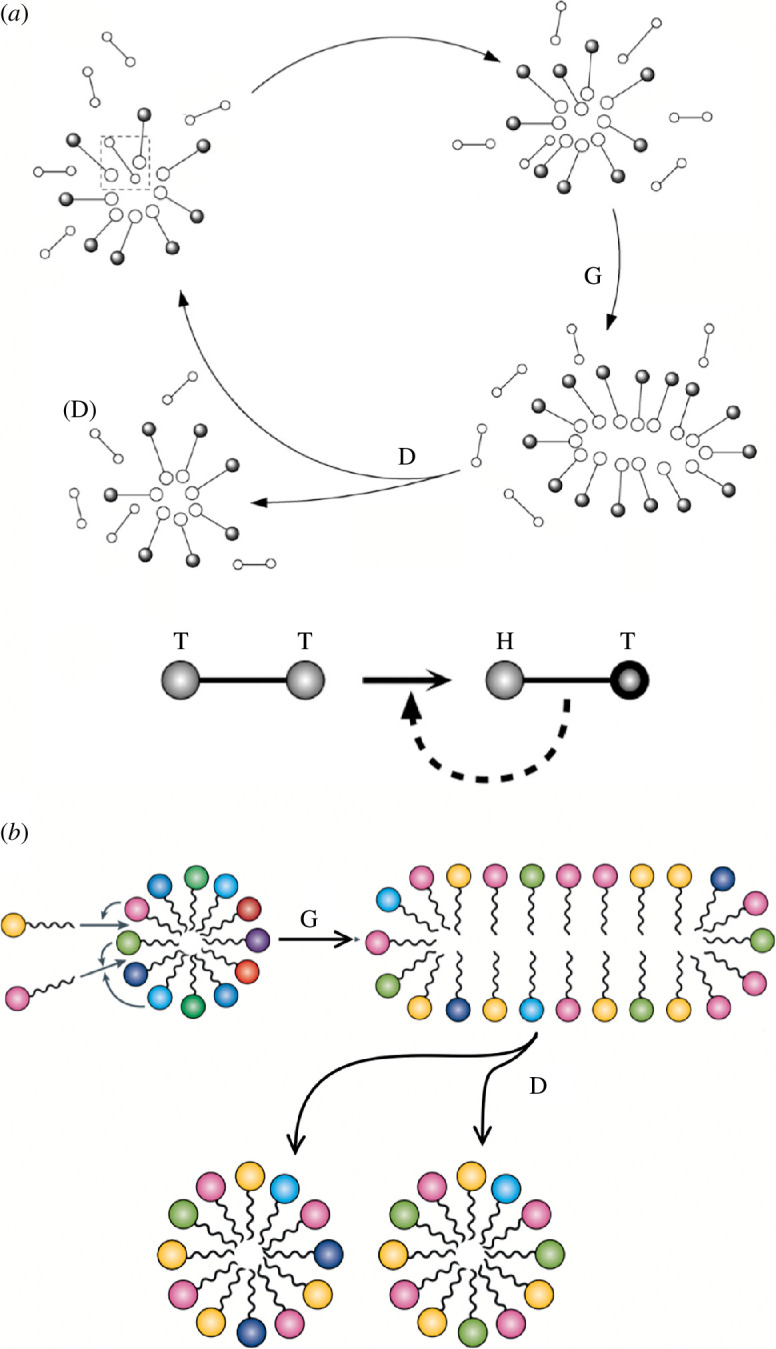
Information-free protocell division. In these two examples, a possible reproduction cycle involving growth G and division D occurs on micelles composed of lipid molecules constantly fed from the environment. In (*a*), these are hydrophobic precursors (indicated as T–T) that react to become a polar amphiphile (H–T), thanks to some reaction (figure adapted from Solé [164]). In (*b*), a similar scenario involves a diverse feeding of lipid molecules forming a heterogeneous aggregate where the recruitment of a particular lipid into the aggregate depends on the compositional information of the aggregate, which lipids already make up the aggregate. In this *lipid world* scenario, the two resulting daughter cells are different (adapted from Kahana & Lancet [171]).

Although most models of cell origins ignore the physical embodiment defined by interacting amphiphiles, any future development will require this component to explain how evolution allowed the reproduction of early cells. However, physical models already show that there are plenty of opportunities. When membrane growth and permeability are coupled to bending energy, a rich space of great morphological diversity is found [165]. Some toy models also include information (figure 4*c*) coupled to metabolism and compartment growth [166]. But models have also shown that such a possibility exists in an information-free context where only metabolism and compartment are present, both in amphiphile-based vesicles [167–169] and micelles [164,170]. Two examples are displayed in figure 5. In both cases, cell division occurs through growth and instability. The first example, simulated explicitly using dissipative particle dynamics [164], displays no evolution. In the second case, each protocellular assembly carries a different set of molecules, and the division rate depends on the *compositional information* associated with the specific combination of surfactants, so this information indirectly modulates the division process that is driven by the relative free energy difference between the mother aggregate and the resulting daughter aggregates.

None of these information-free examples has been observed in our current biosphere. Why? Although the self-organizing properties of information-free soft matter could provide a source of reliable self-replication, which is not present in von Neumann’s formulation, the potential for adaptation and open-ended evolution associated with information-carrying protocells would be difficult to overcome [172]. Thus, we can conjecture that some UC equipped with linear information carriers and exploiting the robustness of self-assembly might have been the expected, perhaps unique solution.

## 5. Multicellularity and development: on growth, form and life cycles

As we mentioned above, the early literature on constraints in development supported the concept that there are limits to the possible in terms of forms and developmental paths [47]. Regarding morphological diversity, multicellular life forms exhibit an enormously rich repertoire of structures. Darwin described this with the famous quote: ‘from so simple a beginning endless forms most beautiful and most wonderful have been, and are being, evolved’ [173]. For a naturalist, this diversity strikes as the most obvious view of the generative potential of life [174]. But is there a truly endless universe of multicellular form, or is the universe of what is actually observed limited due to fundamental constraints affecting the evolution of complex life?

In this section, we address this question by considering three different problems that include different types of constraints, namely (i) the transitions to individuality within the context of the emergence of MC and the emergence of life cycles; (ii) the nature of the complexity classes in pattern-forming dynamics; and (iii) the existence of a physico-genetic toolkit that pervades the emergence of metazoan complexity.

Evidence suggests that the emergence of cells naturally leads to the potential evolution of MC. MC has emerged independently at least a dozen times on Earth, possibly more, though many lineages have been lost to history [175–177]. Laboratory experiments [178,179] and engineered multicellular systems [180,181] suggest that simple MC^10^ is relatively easy to obtain. This is an *evolutionary transition in individuality*. It occurs when replicators, such as cells, form groups that evolve into independent reproducers themselves, causing some loss of autonomy among their parts [182–184] (see also [185–187]). The universal principle is the loss of autonomy in cell-like replicators due to selection acting on groups, favouring traits that enhance group fitness. There is a range of possible MC levels of complexity between strict unicellularity and bona fide multicellular organisms [188]. Crucially, understanding the possible paths to MC requires considering the logic of life cycles [189]. A working definition of MC that encapsulates these features involves the following two properties. (i) Existence. A stage must occur during the organism’s life cycle where a group state is clearly recognisable. (ii) Evolution. Groups must be able to multiply and share heritable information with newly created groups [184]. We note that this definition does not explicitly require groups to be formed of the same species, and indeed, there is a vast array of possible multicellular forms involving multiple species [190–192].

**Figure 6.**
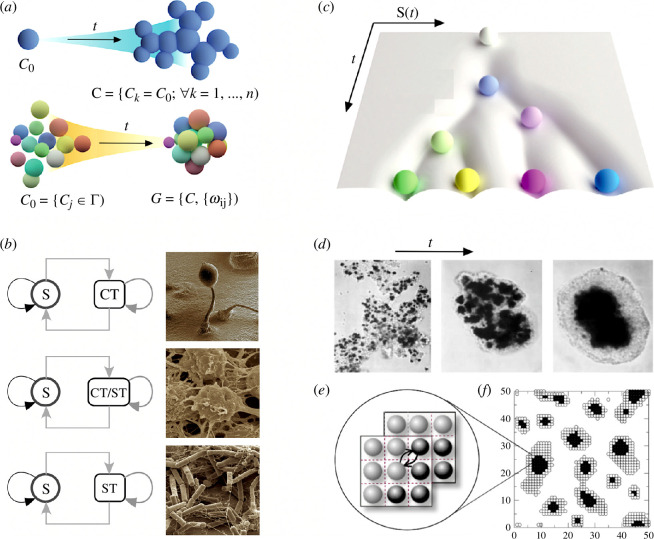
The logic of MC and development. (*a*) At the simplest description level, MC organisms can be assigned to two groups: clonal (upper plot) versus aggregative MC. In the former, a transition $C_{0}\to 𝐂$ occurs after repeated cell divisions with the final population forming a cluster. In the second, the transition is from a set of cells ${𝐂}_{0}$ towards another set (which might contain the same cells and where their states might or might not remain the same) where some interaction matrix $\omega_{ij}$ between cell pairs can be defined. (*b*) MC life cycles can be classified within a well-defined taxonomy where transitions to individuality can be described as graphs connecting (grey arrows) single units (*s*) and aggregates that can be generated by coming together (CT) or staying together (ST) mechanisms, or even the coexistence of both. Shown, from top to bottom, are the life cycles of *Dictyostelium discoideum* (image by David Scharf), *Capsaspora owczarzaki* (from the Multigenome Lab) and *Bacillus subtilis* (image by Arnaud Bridier). (*c*) Within MC organisms, cell differentiation increases organismal complexity and can be described by a succession of symmetry-breaking events on a Waddington landscape. Here, marbles indicate cell types (either transient or final). In (*d*) we show an example of the predictable tissue sorting emerging from a completely disordered cell assembly (adapted from Mombach *et al.* [195]). This can be explained through a simple differential adhesion model (*e,f*) based on cell sorting dynamics [196]. Here the two cell types are indicated by open and filled circles. Adhesion forces are present, and cells can switch their locations in space if the adhesion energy decreases. Panels (*a*) and (*c*) adapted from Márquez-Zacarías *et al.* [197].

What kind of universal logic can be defined here? The first answer to this question comes from the dynamical logic of the problem, which requires the fulfilment of a general principle of biological construction: to create a group, individual units must come together within a finite physical domain and, importantly, deal with the emergence of cheaters [193,194]. In this context, two generic classes of MC can be defined (figure 6*a*,*b*). In the first, MC develops from a single cell $C_{0}$ that generates a clonal assembly through cell division ($𝐂=\{C_{k}\}$), whereas in the second, there is an aggregation of individual cells from a set ${𝐂}_{0}$ that form a cluster that can be defined by a graph $G=(𝐂,\omega_{ij})$ defined by the cell locations and their local interactions. These examples illustrate the following two dynamical processes that can generate MC groups: (i) *stay together* (ST) when, as new units are generated, they keep in close connection with the rest, and (ii) *come together* (CT), which occurs when the units move towards each other. Using these basic mechanisms, it is possible to build a taxonomy of cell cycles [189], three of which are indicated in figure 6*b*, as well as mathematical models that allow exploration of the evolutionary principles [198]. These models reveal that ST can favour the division of labour, while CT allows the exploitation of a combination of units with different properties. Both can be found at every level of biological construction, and their dynamical features define constraints to the possible.

There is another general problem associated with MC: the potential for cheaters to emerge. Parasitic entities are a universal property of evolved complexity (see §7), and parasitic entities can threaten the cooperative nature of MC. The problem is illustrated by groups of microbes that produce some common good (such as a metabolite) that helps the group grow faster than others lacking it. However, within groups, free-riding cheats that do not produce the good usually grow fastest of all [199]. The problem arises from the asymmetry in time scales of growth: MC systems evolve slowly compared to the cells within them, which makes them vulnerable to cellular innovations, as occurs with cancer [200]. How can this problem be solved? One solution to stabilizing MC against cheaters is the evolution of traits that increase cell-level fitness in a group context but come at a cost to free-living fitness. This is enabled by ‘ratcheting’ processes where cells acquire traits that commit them to a group lifestyle. This stabilizes the group and may serve as a universal mechanism that propels multicellular complexity [201,202]. Developmental ratchets would effectively define an arrow of evolutionary time, allowing increases in developmental complexity.

Early forms of MC are typically quite simple and a far cry from the endless forms that so entranced Darwin. Our last consideration here is tied to pattern-forming mechanisms and the emergence of developmental programmes. The rise of complex animals at the Cambrian boundary brought the question of the creative potential of development and the contributions of chance and necessity. In this context, although many components of the MC toolkit were already in place in the unicellular ancestors [203], black swan events might have also predated the origins of animals [204]. An important consequence of the emergence of gene regulation is the possibility of pattern-forming processes across scales, from cells to organisms [205]. The most famous is the Turing instability associated with reaction–diffusion mechanisms [206,207], which exemplifies the role of self-organization as a mechanism to generate spatial order through symmetry-breaking instabilities [208]. These self-organizing phenomena typically involve the interaction between local amplification (due to reaction dynamics) and long-range communication due to diffusion-like processes. This classic picture has been completed with contributions that incorporate mechanochemical interactions [209,210]. Symmetry breaking is partly responsible for the differentiation paths that allow the generation of specialized cell types [197], often represented as dynamical paths on a Waddington epigenetic landscape (figure 6*c*) [211]. On the other hand, the combinatorial power of gene regulatory networks would also suggest that many (perhaps infinite) potential body plans are possible. To address this problem, one needs to consider the nature of the genotype–phenotype mapping, i.e. the map


(5.1) $$\begin{array}{ccc} & \Phi :G⟶F & \end{array}$$

that can be understood as how a given genotype 𝐆∈G is connected with a given phenotype 𝐅∈F. The genotype can, for example, be described by the wiring diagram connecting genes, whereas the phenotype could be the observed spatial distribution of cell states. This formal approach has been used in very different contexts to study shape spaces, including RNA folds [212], pattern-forming gene networks [213–216] and circuits [217]. They all share a remarkable universal pattern of organization, including neutrality, characterized by flat regions where different genotypes have the same fitness [29] (see also Catalán *et al*. [218]). They show high redundancy, with many genotypes corresponding to the same phenotype (thus ensuring stability against mutations) and forming interconnected networks. These properties help explain how genetic variation sustains phenotypic stability and drives evolutionary dynamics while, once again, imposing limits to the space of possibilities [214].

The problem of physical constraints to biological form has been addressed historically [219], in particular by D’Arcy Thompson, who proposed the idea that physical analogies between tissues and foams or liquids could help understand the mechanics of biological form and its changes [220,221]. Despite its limitations [222], new theories inspired in physics have validated many of those intuitions [223–225]. In particular, it has been proposed that metazoan complexity can be generated out of a core set of physico-genetic modules affecting well-defined physical properties such as cohesion, viscoelasticity, diffusion or polarity [226,227]. An example is illustrated by the classical work on cell sorting (figure 6*d*), where a randomly mixed cell population displaying two or more cell types experiences a global arrangement due to differential adhesion energies [228,229]. As an example, we consider three cell types: S=0 (external medium), S=1 (white) and S=2 (black). Cells attach to neighbours more if it lowers the energy per unit area [230]. The energy at position i,j is


(5.2) $$H_{i,j}=\sum_{(k,l)\in \Gamma_{ij}}J_{S_{kl},S_{ij}},$$

where $\Gamma_{ij}$ indicates the coordinates of the set of eight nearest neighbours (Moore neighbourhood) and $J_{S_{kl},S_{ij}}$ indicates the adhesion energy between cell types $S_{kl}$ and $S_{ij}$.

The dynamics is easily defined: choose one cell located in the coordinate r=(i,j) and see if swapping with a neighbour at $r^{\prime }=(i^{\prime },j^{\prime })$ will or not reduce the energy (thus defining spontaneous transitions). If ΔH is the increase of energy in going from the initial to the final state, the probability $P(r^{\prime }|r)$ of swapping is


(5.3) $$P(r^{\prime }|r)=\frac{1}{1+e^{\Delta H/T}},$$

where T is an effective temperature that controls noise. If we iterate this simple probabilistic rule, the system evolves towards a final configuration that can match (in space and time) the observed self-organization towards a stable macroscopic pattern (figure 6*e*,*f*). Single and combinatorial actions of these modules constitute a ‘pattern language’ capable of generating all metazoan body plans and organs, supporting the view that there are strong bounds to the endless forms.^11^

A final word is in force regarding the collective properties of aggregates of cells: through developmental stages, large-scale but precise deformations are possible, thanks to the collective action of cells [234]. Indeed, even the material properties of single cells may live in the continuum; these individual properties are projected at the collective level—for example, the tissue—in a highly non-trivial way [235,236]. Surprisingly, the continuum of cell material properties is projected to the tissue level as a finite amount of different material phases. This result can be theoretically explained, for example, using a slight variation of the energy function proposed in equation (5.2) [236]. Therefore, MC implies collective behaviour and new ways of regulating biological processes across scales. In §8, we will return to the connection between different scales and how this gives rise to the phenomenon of phase transitions.

## 6. Cognitive networks, thresholds and brains

Storing and accessing information is necessary for the emergence of cognitive agents [237,238]. However, decision-making and learning were required for higher cognitive complexity and predictive abilities. In other words, living systems evolved mechanisms that could reduce the uncertainty of the environment [239–241]. Moreover, the emergence of multicellular organisms close to the Cambrian explosion precipitated goal-directed movement and behaviours that required enhanced environmental perception and memory [242].

A general feature of many cognitive systems is the presence of mechanisms that transform analogue signals into digital responses. The need for signal discrimination and the fact that analogue computation is more prone to noise might be two crucial constraints on the evolution of cognition [243–246]. Neurons present a prototypical example of digital information processing and its advantages. Although behavioural patterns existed before neurons, the rapid expansion of neural components enabled novel complex behaviours.

The evolution of neurons [247] and neural circuits [248] allowed life to overcome the limitations imposed by diffusion-limited communication [249]. In both neural and aneural agents, all-or-none behavioural decisions are made using a standard design principle: integrating different inputs is weighted, leading to a threshold-mediated response. This also seems to occur within organisms as well as within cells. Is this a universal design principle?

During the mid-twentieth century, a new wave of computing machines created a technological environment for emulating logic elements akin to those in nervous systems. Pioneering theoretical contributions in mathematical biology by Warren McCulloch and Walter Pitts yielded a groundbreaking revelation: the ability to conceptualize units of cognition (referred to as ‘neurons’) within a logical framework [250,251]. These formal neurons were characterized as threshold units, drawing significant inspiration from the cutting-edge understanding of neural circuits. Unsurprisingly, the McCulloch–Pitts model revolved around the threshold nature of neuron responses and their mathematical depiction [252].

**Figure 7.**
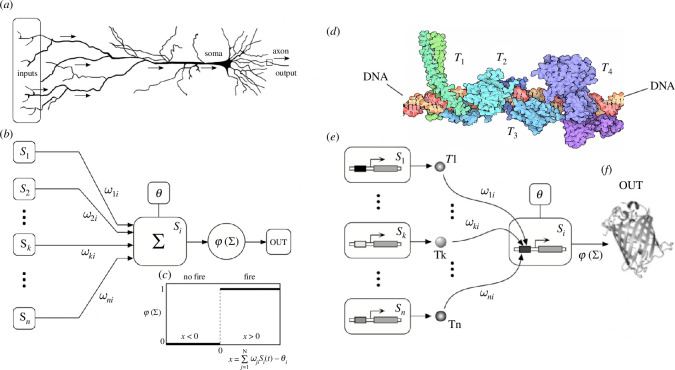
Cognitive networks may exhibit a unique logic of nonlinear response functions. Neurons (*a*) are specialized cells that gather and propagate information in a threshold-like manner. They have a well-defined polarity connected with the input–output signal transmission. The standard McCulloch–Pitts model of a formal neuron (*b*) captures the essence of this transmission in terms of a weighted threshold function, which in its simplest form can be represented as a binary on-or-off response (*c*). Other information transmission systems, like genetic networks, are usually modelled similarly (*d,e*). For example, TFs ($T_{i}$) are proteins expressed by some genes that bind to DNA (*d*; image by David Goodsell) and regulate the expression of other genes. The input–output diagram in (*e*) is analogous to the neural counterpart, and the corresponding response functions are also highly nonlinear threshold functions. The outcome of these interactions can modify the expression of a given protein (*f*) or set of proteins.

The illustrative depiction of the McCulloch–Pitts model’s conceptual framework is outlined in figure 7*a*,*b*. The presented ‘formal neuron’ (figure 7*b*) operates as a simple Boolean system, with its state assuming one of two values: $S_{i}\in \Sigma \equiv \{0,1\}$ (an equivalent description of neurons as spins, $S_{i}\in \Sigma \equiv \{-1,+1\}$, is sometimes employed when studying neural circuits using techniques from statistical physics). These two states typically correspond to neurons at rest (inactive) or in the act of firing (sending signals to other neurons). In response to incoming signals from a set of N pre-synaptic units, formal neurons exhibit sudden activation if the weighted sum of inputs surpasses a threshold [253,254].

While activation follows an all-or-nothing principle, the weights, denoted as $\omega_{ij}$, are continuous and indicate the impact of the state of pre-synaptic neuron j on post-synaptic neuron i, thereby modelling the strength of connections. These weights can be either positive or negative, allowing for the implementation of excitation and inhibition. In the McCulloch–Pitts approach, the integration of incoming signals by the post-synaptic neuron $S_{i}$ is expressed as


(6.1) $$\begin{array}{r} S_{i}(t+1)=F\left(\sum_{j=1}^{N}\omega_{ij}S_{j}(t)-\theta_{i}\right), \end{array}$$

where the additional parameter, $\theta_{i}$, establishes the neuron’s threshold. The nonlinear step function F(x) yields a value of 1 if its argument is positive and 0 otherwise. Consequently, neuron $S_{i}$ fires when the weighted sum of pre-synaptic inputs surpasses its threshold. The introduced nonlinearity through F(⋅) enforces the all-or-none neural response.^12^

McCulloch and Pitts demonstrated a pivotal insight: combinations of formal threshold neurons can be used to construct any logic Boolean circuit. This implies that, at least in their Boolean representation, brains could execute the same logic operations as computers. The McCulloch–Pitts model and its descendants have influenced the further development of artificial neural networks. Beyond the single-unit design discussed here, artificial neural networks were inspired by another seemingly universal design principle associated with cortical architecture [255]: the presence of multiple processing layers (such as those found in the visual cortex). Are there alternative approaches to designing cognitive networks that do not rely on threshold-like units?

Interestingly, the basic McCulloch–Pitts design is common to many other living systems, including gene regulatory networks [256–259], immune networks [260–262], collective intelligence [263–267] or some aneural systems, such as quorum-sensing decisions in microbial communities, where an explicit equivalence has been defined [268]. These examples share an essential feature that departs from standard neural networks: they are ‘liquid’, meaning that the parts (proteins, bacteria or ants) move in space and that there is no stable, hardwired connectivity among pairs of individuals [269,270]. Do these systems follow a different logic scheme from the integration-threshold motif?

We illustrate this equivalence by considering gene regulatory networks, one of the best-studied examples. In this case, expression levels of transcription factors (TFs), to be indicated as $\left[T_{i}\right]$ (intracellular concentration) of different TF ($T_{i}$ in figure 7*d*) change following a dynamical model [271]


(6.2) $$\frac{d[T_{i}]}{dt}=-\lambda_{i}[T_{i}]+G\left(\sum_{j=1}^{N}\omega_{ij}[T_{j}](t)-\theta_{i}\right),$$

where $\lambda_{i}$ is the degradation rate and G is a threshold-like function that is a consequence of the order of the molecular nature of interactions between DNA and TF. More precisely, because TF typically forms dimers, the nonlinearity associated with dimerization automatically implies cooperative, threshold-like responses [272,273]. Here, the weights $\omega_{ij}$ encapsulate diverse factors influencing the binding of TF.

What are the consequences of employing polarized, threshold-like elements in line with McCulloch–Pitts logic? This approach may facilitate the early development of multilayer processing structures, including interneurons as a crucial innovation, re-entrant closed loops and memory circuits capable of learning [274,275]. Are loops and multilayer architectures a convergent evolutionary design? A positive answer is suggested by the comparative study of neural networks in invertebrates and vertebrates [276,277]. Despite evolving along separate branches, these groups exhibit similar network topologies. This resemblance is observed in layered neural networks described by Ramon y Cajal for insect and cephalopod visual systems [278,279]. It has also been suggested that neural circuits with re-entrant loops are crucial for complex cognitive tasks [280]. One implication of these shared architectures is the potential for convergent minds shaped by evolution [281–284].

## 7. Ecology: inevitable parasites and functional trees

As the ecologist Ramon Margalef said [285], there is a ‘baroque of nature’: there are so many species that an inevitable question is ‘why so many?’ Presumably, an alternative, much simpler prokaryotic biosphere could fulfil all biogeochemical functions of ecosystems [286]. In Margalef’s view, even though physical limits bind the organization of ecosystems, there is plenty of room for the possible within these limits. The emergence of variations and the feedback between species and their environments might explain communities’ regularities and diversity. And yet, here again, the ecological literature is full of examples of systems where continuous features can be effectively discretized into a small number of categories.

Two basic components of this discretization are involved. First, there is a well-known classification of the interactions between two species into combinations of neutral, positive or negative exchanges [287]. From this set, {0,+,−}, different pairs can be derived


{{0,0},{+,+},{−,−},{+,0},{−,0},{+,−}}

with each pair mapping neutralism, competition, predation or parasitism, among other possibilities.^13^ Despite other features affecting each species at play, further classification within each class reveals again a discrete repertoire.^14^ Such an approach has been made, for example, within parasites, showing that, despite the enormous diversity of habitats, hosts, sizes or shapes, their diversity of strategies largely transcends phylogenetic boundaries [290].

Are there alternatives to these ecological network architectures? Would ecological webs in other planets (with different energy flows and chemical diversity), display different structural patterns? [291–293]. All these interactions occur (unless under controlled conditions) in a given environmental context where limited resources are available.^15^

**Figure 8.**
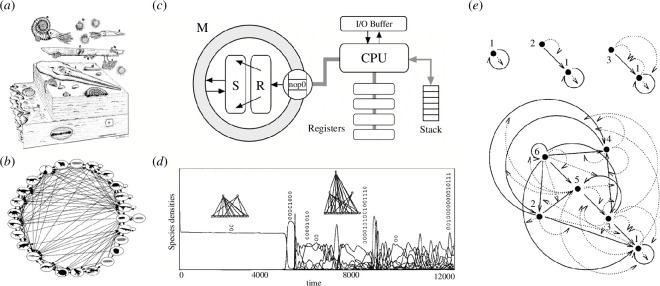
Universal patterns in ecosystems and digital ecologies. Despite their huge diversity, ecosystem architectures are identified in current and fossil communities (*a*) and the ecological network (so-called ‘paleo food webs’) reconstructed, as shown in (*b*) for a fossil ecosystem before the K–T extinction. One approach to the evolution of these ecological networks relies on digital versions of species and their interactions. An example is shown in (*c*), where the virtual CPU of the Tierra system is summarized (adapted from Adami [300]). Here, S and R stand for the Slicer and Reaper queues, which introduce rewards and ageing. It is possible to evolve food webs (*d*) with a discrete number of layers using evolutionary dynamics on bit strings encoding game-theoretic models (adapted from Kristian & Nordahl [301]). Algorithmic reactors allow evolving interaction networks and see sequences of increase in complexity, as illustrated in (*e*), where different ‘species’ are indicated as filled nodes, where interactions can happen directly (continuous arrows) or through an intermediate operator rule (adapted from Banzhaf [302]). The evolved networks always include parasitic interactions.

Here enters the second component of discretization because the stability properties of these networks largely determine the separation between what is possible and what is not [295,296]. Due to these stability constraints, we can predict that some communities are impossible to observe (cannot be realized). Moreover, despite the enormous variation of climate and resource conditions, ecosystems display essentially the same properties, from Antarctica to the Sahel [297]. We can also reconstruct fossil ecosystems, the so-called ‘paleo food webs’ [298,299] (figure 8*a*,*b*), and again find universal patterns shared with modern ecological networks [303].

By avoiding the actual embodiment of biological agents and going down to their algorithmic description, *in silico* experiments support the presence of constraints in the logic of possible ecosystems. The first historical attempt to simulate an evolving digital ecology was made in the 1950s by Niles Barricelli, using the MANIAC computer at Los Alamos National Laboratory, as a digital environment [304,305], who wanted to test the theory of *symbiogenesis* [306]. These simulations were based on a grid of cells, each representing an individual organism. The organisms, defined as binary strings, could reproduce, mutate and compete. Complex interactions emerged but with an important characteristic: parasites rapidly evolved, jeopardizing the potential for creating diversity and evolving complexity. As with natural systems, they were a seemingly inevitable component.

Barricelli’s ideas were forgotten until the days of artificial life in the 1980s [307–309]. Two crucial developments attracted renewed attention to the evolution of virtual agents: (i) the formalization and propagation of computer viruses and (ii) the creation of computer ecologies. Computer viruses can be seen as an inevitable outcome of computer networks interacting with data storage and transmission processes. Fifty years after Kleene’s theorem, self-replicating infectious programmmes were designed to infect local network servers. From a meagre presence in 1990, DOS-based computer viruses skyrocketed to over 10 000 by 1996. This surge prompted an arms race between new viruses and programmers [310], and their spread was shown to follow the epidemic nature of their real counterparts [311]. This is a perfect illustration of a convergent pattern that is shared between living and artificial systems. What about other ecological interactions?

Ecologist Tom Ray designed the program Tierra on a laptop, creating a virtual architecture in the host computer’s memory with its instructions and memory space [312,313]. Each digital organism has a genetic code that dictates its behaviour and features. Replication introduces mutation through faulty ‘genetic code’ copying, while selection happens via competition for memory and CPU resources. Tierra unexpectedly displayed diverse ecological interactions due to the digital selection process. After the growth of shorter, faster-replicating programmes, parasites emerged, needing other programmes for reproduction. Hyper-parasites and immunization mechanisms followed. Recombination (primitive sex) emerged in response to threats, leading to social behaviour through programme cooperation. Other simulations of evolving ecosystems confirm the generative potential of artificial life systems [300,314–317]. Despite differences, the convergence in interaction classes suggests that the discrete set of possible interactions is standard in both digital and natural worlds. Some artificial life models use game-theoretic approaches with coded strategies in a digital genome. Complex patterns emerge, including coevolution, extinction (punctuated equilibrium [318]), cooperation [319] and ecological networks involving trophic levels [301] (figure 8*d*).

These patterns can also be explored using dynamical models that have been used traditionally within the context of artificial chemistries [320,321] and deterministic chemical reaction dynamics [322]. The latter involves systems of coupled differential equations with higher-order nonlinear terms. One standard formulation is


(7.1) $$\frac{dN_{k}}{dt}=\sum_{i=1}^{s}\sum_{j=1}^{s}\alpha_{ij}^{k}N_{j}N_{i}-N_{k}\Phi (N),$$

where $N_{i}$ is the population of type i, $𝐍=(N_{1},...,N_{s})$ is the concentrations vector representing s different types, and each sum includes the different potential reaction events leading to the production of type k, i.e. the bimolecular reactions


(7.2) $$\begin{array}{ccc} & i+j⟶i+j+k & \end{array}$$

along with their associated rates $\left\{\alpha_{ij}^{k}\right\}$. These are kinetic models grounded in reactions assuming random molecule collisions. The term Φ(𝐍) stands for a dilution flux that keeps the condition $\sum_{j}N_{j}=1$ of constant population.^16^

How can we introduce evolutionary rules and a context where functionalities are represented? Some particular formulations of these models describe Darwinian dynamics in molecular reaction networks [323] (see also [324]). This occurs when mutations are introduced as one specific reaction class, whereas mean replication is a fitness measure. A finite set of resources provides the source of selection.

The different types of entities whose evolution is described by equations such as equation (7.1) do not need to be restricted to chemical species. An *algorithmic* reaction system can be built using abstract symbols, binary strings, numbers or even proofs [302,321]. In Fontana’s *Alchemy* (for algorithmic chemistry), the emergence of novelties is possible by assigning to object interactions the formal properties of functions operating on data [325,326]. In this case, objects act based on symbol binding and λ-calculus rules, where one object functions on another as an argument, resulting in a new object. For two objects f(x),g(x)∈F, an interaction leads to a composed object f[g(x)], creating a new object h=ϕ(f,g)∈F. Interactions define a mapping: ϕ:F×F⟶F, representing any computable function, with constraints from underlying semantics. If $P=2^{F}$ (power set of F), an additional mapping M:P⟶P describes the allowable ‘collisions’. In the resulting *Turing gas* model, dynamics enable the compositional generation of new objects and the formation of complex interacting object networks, including hypercycles [327–329]. This kind of algorithmic chemistries can lead to complex networks of interactions (starting from catalytic cycles) that also contain parasites (figure 8*e*).

**Figure 9.**
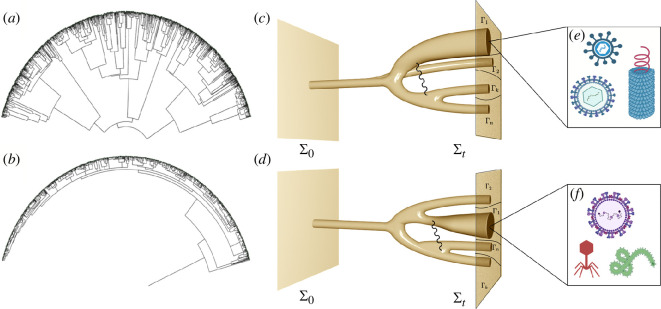
Parasites and functional evolutionary trees. Digital ecologies generate well-defined, qualitative classes of agents that we can identify as parasites, predators or natural counterparts associated with a discrete set of constraints in the repertoire of potential functional roles. In (*a*,*b*), we display the evolutionary trees of an artificial life implementation using *Avida*, where parasites are present (*a*) or suppressed (*b*). The resulting phylogenies indicate that parasites promote diversity (adapted from Zaman *et al*. [333]). The convergent patterns exhibited by ecosystems are illustrated using a diagrammatic representation of two ‘runs’ (*c,d*) of an abstract world, starting from an initial set of species $\Sigma_{0}$ and looking at a final set of species $\Sigma_{t}$. Branches appear each time a new functional class emerges. Although the branching patterns might differ, we conjecture that *functional trees* will branch into the same qualitative, discrete classes $\Sigma_{t}=\underset{k=1}{⋃}\Gamma_{k}$. A thick branch associated with parasites will always be present in both cases, with diverse (but common) solutions indicated on the right (*e,f*) using examples from extant viruses (images generated using BioRender). Each time viruses emerge, they will influence the dynamics and even the emergence of new branches, as sketched by the wiggled lines.

The widespread presence of parasites in both artificial and natural communities suggests that they are a universal outcome of the evolution of complex adaptive systems [330–332]. *In silico* coevolution models [333,334], *in vitro* evolution [335,336] and genomic analyses [337,338] have shown that parasites actively promote diversity and evolvability, as illustrated by the phylogenetic trees in figure 9*a*,*b*. Here, the presence (figure 9*a*) or absence (figure 9*b*) of parasites dramatically alters species richness, consistently with their role in natural communities [339]. A construct that we term *functional* evolutionary trees can be introduced to provide a unified picture of these case studies. Consider a given evolving biosphere (living or artificial) where novel forms of interactions among agents emerge. Some forms result from adaptations offered by available energy pockets (such as those that occur with parasites), while major innovations mediate others. We could build an evolutionary tree (figure 9*c*,*d*) where we cluster together those species that share a common set of defining ecological behaviours.

Results from artificial life models, as discussed above, suggest that there is a finite set of possible classes of interactions, namely a set $\Gamma =\{\Gamma_{0},\Gamma_{1},...,\Gamma_{n}\}$, where each $\Gamma_{j}$ contains species sharing the same qualitative attributes. Initially, we start from a set of species $\Sigma_{0}$ containing a single homogeneous population of individuals of a single class $\Gamma_{0}$ (such as Ray’s initial set of programmes). Due to evolutionary branching, new classes emerge up to a given time where several clusters (classes) $\Gamma_{k}$ are found in $\Sigma_{t}$, where the diversity of each class is likely to depend on environmental variables $\left\{e_{j}\right\}$. We conjecture as a result of evolutionary dynamics, the classes form a partition of the final set of species $\Sigma_{t}$, so that


(7.3) $$\Sigma_{t}=\overset{n}{\underset{k=0}{⋃}}\Gamma_{k}(e_{1}^{k},...,e_{m}^{k});\;\;\;\Gamma_{k}\cap \Gamma_{j}=\emptyset \;\;\forall k\neq j.$$

Commonalities across different runs would reflect the convergent dynamics of our evolving systems. Specifically, how trees branch over evolutionary time would be somewhat path-dependent (the kinds of parasites might differ; figure 9*e*,*f*). Still, the final structure of ecological networks would include the same set of ecological roles.

Another perspective on ecology follows by considering observed regularities and what they might tell us about life [43,44,340,341]. One of the most powerful recent approaches to ecology connects allometric scaling laws with fundamental physical constraints [43,104,342]. Recently, such approaches to ecology have been extended to create novel biosignatures [340] and to suggest that the logic of cellular metabolism may be universal [341].

## 8. Phase transitions and critical states

Some of our previous case studies are deeply connected with the emergence of major innovations in evolution. These so-called *major evolutionary transitions* [343] refer to critical points in the history of life on Earth where new levels of biological organization (cells, MC or language, to cite a few) and complexity emerged. It has been suggested that these transitions, which imply a marked shift from a given qualitative level of organization to a new one, can be mapped into the concept of phase transitions from statistical physics [13,58,61,344,345]. Because of the nature of these transitions, they are likely to be relevant in our understanding of macroevolutionary processes, ranging from punctuated equilibrium [318] to the hierarchical nature of evolutionary change [346,347] and the role that constraints play in setting transition points [105,348,349].

The theory of phase transitions was developed within physics over roughly 90 years spanning most of the twentieth century [350–356], delivering a fully formalized and consistent theory of phases and the transitions separating them. The results have strongly influenced efforts to build a ‘systems science’ approach to complexity in this area [357,358]. As discussed in §1, the historical nature of living matter seems to play a secondary role when looking at the fundamental logic of its organization. How much of physics constrains the possible, and what is the relative role of contingency? Interestingly, the two components are not exclusive. This is known as *symmetry breaking* [359,360].

**Figure 10.**
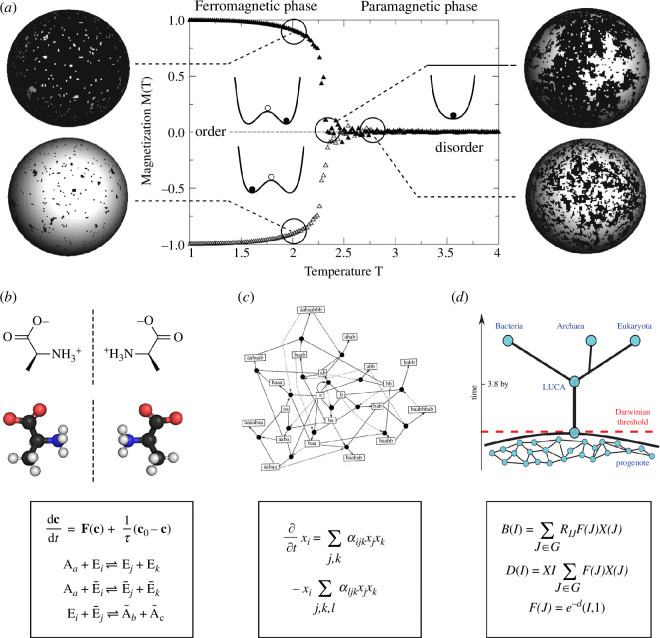
Phase transition in physics versus evolutionary transitions. In (*a*), the phase transition associated with the two-dimensional Ising model is shown, displaying the average magnetization (the order parameter) $M(T)=\sum_{i}S_{i}$ (i.e. a sum over all spins $S_{i}\in \{-1,+1\}$) against the temperature (the control parameter). The snapshots are obtained by indicating ‘up’ (+1) and ‘down’ (−1) spins as black and white squares, respectively. Two possible ordered configurations are obtained for temperatures lower than the critical $T_{c}$, associated with a symmetry-breaking phenomenon. For $T>T_{c}$, a disordered phase is present, with an essentially random arrangement of spins, lacking spatial correlations. A critical point $T_{c}$ separates these two phases. The inset curves represent the macroscopic potential function derived from a mean-field approximation. Open and filled circles stand for unstable and stable states, respectively. In (*b,c*), we display three examples of models of evolutionary innovations that exhibit some phase transition, along with some of their underlying mathematical descriptions. These are (*b*) the emergence of homochirality (figure from Wikipedia [364]), (*c*) the emergence of autocatalytic cycles out of random chemistry of reacting species (figure redrawn from Farmer *et al*. [365]) and (*d*) the collective evolution of genomes as described by the transition from horizontal to vertical genetic transition (figure from Goldenfeld *et al*. [59]).

We can illustrate this concept using one particularly successful and influential model: the so-called *two-dimensional Ising model* [361–363]. It is a discrete model with simple rules to explain phase transitions in ferromagnetic materials (see figure 10*a*). In this model, a two-dimensional lattice represents a magnet with units $S_{k}$ (k=1,...,N) having two possible states (spins): up ($S_{k}=+1$) and down ($S_{k}=-1$). Quantum mechanics suggests the lowest energy state occurs when adjacent atoms share the same spin. The global magnetization $M=\sum_{k}S_{k}$ depends on the difference between the total number of up and down spins ($N_{+}$ and $N_{-}$). Spins align at low temperatures ($T<T_{c}$), resulting in ordered states in what is known as the ferromagnetic phase, where |M|>0. Since two possible, completely symmetric ways of alignment are at work, the symmetry must be ‘broken’. Above $T_{c}$, disorder prevails, with M≈0. Here, the temperature T is the *control parameter*, whereas the magnetization M is the so-called *order parameter*. As T is tuned, two distinct phases with marked macroscopic properties are observable, separated by a well-defined critical point $T_{c}$.

The Ising model considers an energy function H, based on the microscopic interactions between magnet units:


(8.1) $$\begin{array}{ccc} & H(S_{1},...,S_{N};J)=-\frac{1}{2}\sum_{\left\langle i,k\right\rangle }JS_{k}S_{i}. & \end{array}$$

The coupling constant J defines the strength of the interaction between nearest neighbours ⟨i,k⟩ within the lattice of magnets. Dynamical changes in the orientation of the magnets are introduced using transition probabilities that depend on temperature and neighbour spins. The transition probability is defined using a temperature-dependent function


(8.2) $$\begin{array}{ccc} & P[S_{i}\to -S_{i}]=\frac{1}{1+e^{\Delta H/k_{B}T}}, & \end{array}$$

where ΔH is the energy change associated with a spin flip.

The Ising model has been shown to predict experimental observations of magnetic systems near $T=T_{c}$ with high accuracy. This is an important fact since the model ignores most of the microscopic details of the real system, keeping only the symmetry of the interactions, demonstrating universality in critical phenomena theory. Multiple extensions have been proposed, and the number of applications is huge, even considering simple, mean-field versions of the model.^17^

Indeed, beyond ferromagnetism, the two-dimensional Ising model and its extensions have been used in many contexts within complex systems. This includes its equivalence to Eigen’s quasispecies model, thus allowing mapping the error threshold as a phase transition [367–369], cell membrane responses [370], multicellular assemblies [371,372], spatiotemporal changes in rainforests [373], universal models of complexity [374] or large-scale functional brain dynamics [375]. We mention this disparate set of examples because, in all cases, it was possible to accurately understand complex phenomena on a qualitative and quantitative basis.

Several well-known examples of evolutionary innovations seem to be associated with a symmetry-breaking event. These include, for example, the transition from ‘pre-volution’ to evolution [376], natural selection [377], the universality of intermediate metabolism [86,98] as well as the origin of chirality (figure 10*b*) [378–380] as a mechanism to favour one of the two possible (symmetric) solutions through an amplification phenomenon: the final choice would be a historical accident. Other transitions involve the jump to novel properties associated with increased network connectivity. This would be the case of the emergence of autocatalytic cycles out of random chemical reactions (figure 10*c*) once the number of possible reactions crosses the percolation threshold (a non-equilibrium phase transition) [381–384]. Similarly, the collective evolution of the genetic code might have also resulted from a phase transition (figure 10*d*). In this case, the combination of threshold conditions for forming phases and their subsequent robustness in supporting the selection of higher-order organizations was invoked to explain the assignment of amino acids to codons in the genetic code [385]. The insight of this argument, first articulated by Woese in 1967 [386], was that although a *highly reliable* code could function adequately with codon assignments that were a frozen accident as Crick suggested [387], the actual genetic code implemented in molecular machinery is subject to errors even in extant, highly evolved life, and must have been much more error-prone in the earliest eras of ribosomal translation and genome replication and transcription.

Along with the potential connection between evolutionary innovations and phase transitions, critical points play another role. While the previous examples deal with crossing the boundaries from one phase to another, some innovations are tied to the evolution towards critical states. Criticality (sometimes also called ‘the edge of chaos’) usually refers to the state where the system is poised between two phases: an ordered phase (high order parameter) and a disordered phase (vanishing order parameter). For instance, criticality occurs in the two-dimensional Ising model when $T=T_{c}$. The idea that criticality might have several desirable properties has been advanced by different scholars within the context of nonlinearity and chaos [388], computation [389], genetic codes [390], virus evolution [391], virus–immune system coevolution [392], neuroscience [393,394] or ecology [395,396]. There are several good reasons for critical states playing a key role in living systems: information transfer becomes optimal at criticality [397,398] and sensitivity to external signals is maximized [399].

One hallmark of these transitions and their macroscopic emergent phases is their significantly lower^18^ range of variation compared to the microscopic configurations that produce them. While the possible microscopic configurations can increase combinatorially with system size, the self-reinforcing patterns that emerge can take far fewer forms, sometimes only finitely many, leading to a substantial reduction in the degrees of freedom for the system’s potential states [402]. From a developmental perspective—as we already outlined in §5—the existence of material phases at the tissue scale defines a low-dimensional scenario of potential tissue properties and structures with obvious regulatory advantages [403–405]. For example, it has been reported that the embryonic tissue in its early stages lies close to the fluidization critical point, enabling the tissue to *melt* and thus induce a strong deformation to *solidify* again, to fix the morphogenetic changes [406]. This discrete nature comes hand in hand with side effects: cancer progression, in some stages, is more efficient thanks to the fluidization/rigidification of the involved tissues [407]. In addition, huge fluctuations due to the proximity to the critical point may represent a serious drawback for the precision of tissue development [406]. From an evolutionary perspective, these robust properties can be seen differently: phase transitions define a clear boundary separating two qualitative behaviours, with each phase characterized by a few fundamental parameters. Despite their differences, systems that undergo these transitions will exhibit some fundamental common laws of organization. This phenomenon is known as convergence.

One final point is related to the nature of the possible laws that could drive the increasing complexity of living entities. By default, it is assumed that natural selection is the dominant process at work, although alternative scenarios based on persistence-level selection (lacking replication) need some consideration [408]. On the other hand, our previous examples suggest that self-organization and emergent phenomena are as relevant as selection [382]. Is that the case? Could other types of dynamical processes also account for the generation of complexity? Manfred Eigen proposed that natural selection is a phase transition in itself [377], and some authors suggest that this is likely to be a universal principle [409].

This section concludes with an idea suggested by several previous works [61,376,410]: deep constraints also limit the possible kinds of evolutionary laws ruling the biosphere. These would include (along with the thermodynamic laws discussed in §2) (i) an inevitable requirement for autocatalysis [411] as a mechanism for population amplification, (ii) the emergence of molecular heterogeneity as a pre-condition for population dynamics, and (iii) the phase transition to evolution from a non-Darwinian to Darwinian biosphere once some given interaction thresholds are achieved. Future work should consider how a theoretical framework can be defined to prove the uniqueness of natural selection as the expected generative force that drives biological complexity.

## 9. Discussion

Are there multiple alternative ways to generate living complexity? Predicting potential universal features of living systems, such as those resulting from constraints in the examples discussed in this paper, poses several significant challenges. Given the lack of multiple alternative scenarios, how can we address the logic of life? This question makes particular sense under a view of evolutionary dynamics as a highly path-dependent process. If life (however defined) can unfold in highly dimensional spaces that cannot be fully explored, there seems to be plenty of room for divergent designs. However, the existence of constraints in those spaces of the possible might profoundly change this view. The implications have been discussed in the context of possible life, from chemical constraints to phenotypic convergence in an alternative biosphere. Still, they are likely to be relevant in understanding the limits of bioengineering design. In this context, many of the questions raised in our work have been the target of dedicated efforts within the field of artificial life [300,412–414]. The ‘surprising creativity’ of digital evolution has been an extraordinary source of inspiration and understanding [415].

In this paper, we conjecture that there are constraints that limit the logic of life across scales. To this goal, we provide a set of arguments suggesting that computational, physical and dynamical constraints profoundly limit the design space of possible living systems. The examples presented here have been supported by different but complementary arguments (computational, ecological, chemical, etc.). Our list (which by no means is intended to be exhaustive) includes the following.

— Internal entropy-reducing processes characterize the thermodynamic logic of living systems. Such processes are enabled by coupling processes that produce greater entropy in the environment, likely in the form of generated heat. Life is also expected to store and employ energy intermediates to drive internal processes and to decouple from environmental conditions, thereby attaining a degree of thermodynamic autonomy. Finally, the internal metabolic process will be organized around cyclic transformations.
— Linear heteropolymers formed by sets of units (symbols) having near-equivalent energies are the expected substrate for carrying molecular information. They allow the exploration of vast combinatorial spaces, and the physical constraints associated with linearity might pose severe limitations to the repertoire of possible monomer candidates.
— Closed cell compartments equipped with a von Neumann replication logic are needed for self-reproducing living forms capable of evolution. The compartment allows the concentration of required molecules and defines a boundary between internal and external environments connected through a membrane that can play a part in the constructor roles by exploiting physical instabilities. Such a closed container can be achieved using a specific class of molecules (the amphiphiles) and is thus constrained to a subset of chemical candidates.
— MC allows the emergence of new kinds of organization out of simpler units. One universal pre-condition for this innovation is the presence of some physically embodied process that guarantees the closeness of cells. While the group provides mechanisms of efficient collective reproduction, these new units of selection (from cell clusters to organisms) need to deal with cheaters through ratcheting. The potential diversity of basic morphological designs might be strongly constrained by a finite number of physico-genetic motifs, whose combinations might generate the whole repertoire of basic developmental programmes sharing deep common morphological motifs.
— Beyond information coding on coded strings, cognitive systems require threshold-like units that allow reliable integration and decision-making. Complex cognition has been unfolding by evolving different (but formally equivalent) circuits based on threshold functions that integrate surrounding signals. In multicellular systems, this means evolving cells that display polarization and provide the means for rapid sensing and propagation of information. Because of these features, complex cognition might have been constrained to evolve towards multilayer systems.
— Ecosystem architectures are deeply constrained within a finite set of possible classes of ecological interactions. Current and past ecosystems reveal such a discrete repertoire of possibilities, and *in silico* models of evolving ecologies support this constrained repertoire. Among other regularities, the widespread presence of parasites suggests that they are an inevitable outcome of complex adaptive systems.

Each particular case questions us about different aspects of the evolutionary logic of biocomplexity. Each of the conjectures requires a rigorous formulation of the hypotheses and, in most cases, the consideration of diverse fields of analysis, from information theory and statistical physics to astrobiology and evolutionary biology. Solutions may require the development of new conceptual frameworks beyond the boundaries associated with each field. Furthermore, the problems under consideration are part of a hierarchy in which certain properties at one scale can affect (or be affected) by those at the next scale.

Does the notion of fundamental constraints for life eliminate the potential for surprises? Certainly not. Concentrating on logical structures ignores an essential aspect of multicellular life: the stunning diversity of morphological, anatomical and physiological adaptations that evolved in response to environmental factors. However, if our proposed constraints are universal, the logic of life elsewhere is likely to be quite familiar.

## Data Availability

This article has no additional data.

## Footnotes

^1^
Those estimates are based on evidence from the fossil record that reveals a sharp transition, whereas molecular clock studies (where divergence times are obtained from sequence comparison) locate the origin of animals at about 780 Ma (see [22] and references therein).

^2^
The mapping between genes and phenotypes is poorly captured by the linear connection. The nature of the genotype–phenotype mapping [29] is highly compositional and algorithmic, reflecting the inevitable and essential interdependence of the molecular components that constitute organisms and enact their physiology and development.

^3^
In statistical physics, an ergodic system is one which, over sufficiently long timescales, explores all possible microstates that are consistent with its macroscopic properties. Mathematically, for any physical observable A(𝐪), where 𝐪 is the microstate that specifies all of the system’s coordinates, the long-time average of A(𝐪) converges to the ensemble average, so

$$\lim_{T\to \infty }\frac{1}{T}\int_{0}^{T}A(q(t))\;dt=\langle A\rangle .$$

In intuitive terms, an ergodic system explores (over time) all possible ways it can exist. It suggests that, given enough time, the system will visit all the available configurations or states.

^4^
In non-equilibrium thermodynamics [78], the rate of entropy production per time τ can be expressed as an integral

$$\Delta S_{tot}/\tau =\int_{V}\sigma \;dV$$

for a system of volume V, with σ indicating the specific entropy production rate, to be written as

$$\sigma =\sum_{k=1}^{n}X_{k}J_{k},$$

where we have a set $\left\{X_{1},...,X_{n}\right\}$ of ‘forces’ (such as temperature gradients or chemical affinities) and their conjugate fluxes $\left\{J_{1},...,J_{n}\right\}$ (such as flows of heat or reaction rates).

^5^
As mentioned above, thermodynamic principles drive the formation of metabolic cycle emergence in living systems, thereby creating interconnected information flow cycles. A generalized information-theoretic framework [111] explains different kinds of structures, using a variational principle to balance diverse information pathways and optimal flow [112]. These network approaches further expand the relevance of constraints at various levels [113,114].

^6^
Once helicases unwind the double-stranded DNA helix.

^7^
To a large extent, the complementary nature of DNA is what allows enormous flexibility in building structures and machines. A given strand will match only its complement and weakly interact with other sequences. In this way, many components can be assembled together in two or three dimensions while keeping the control on interaction strengths between every pair of elements.

^8^
A distinction needs to be made between replicators and reproducers. As suggested by E. Szathmary, reproduction of a cell implies making a copy of the whole that is not limited to the genome, in contrast with viral particles or other putative ‘naked’ molecular replicators in early life.

^9^
Some crucial insights into this problem were advanced within the context of Turing machines. If a universal Turing machine can have access to its own code (a form of self-description), it is possible to show that one can re-write any program so that it will print out a copy of itself before it starts running. In formal terms, for any Turing machine T, there exists a $T^{\prime }$ such that $T^{\prime }$ prints out a description of $T^{\prime }$ on its tape and then behaves in the same way as T. Conceptually, this is reminiscent of the UC, which makes a copy of the Instructions and then carries out the Instructions. The mathematician Stephen Kleene first developed these ideas in his second recursion theorem [144,145].

^10^
Simple MC (SMC) refers to a form of multicellular organization where cells form clusters or colonies with limited differentiation and coordination. Unlike complex multicellularity, where cells exhibit specialized functions and are organized into distinct tissues and organs, SMC involves groups of similar or identical cells working together, often with minimal communication or structural integration.

^11^
In some cases, as occurs with fractal branching patterns in plants [231] or growing corals [232], there is a set of generative rules (that sometimes can be expressed in terms of grammar) that successfully reproduce the whole repertoire of forms, which can often be classified within a parameter space in terms of separated phases. For intriguing arguments that fractal growth with environmentally regulated parameters governed the Ediacaran fauna that may have been a transition stage to non-self-similar development, see [233].

^12^
Alternative implementations may employ smooth step functions, such as

$$F(x)=\frac{1}{1+e^{-\beta x}},$$

where β (an inverse temperature) governs the smoothness, converging to the discontinuous step function as β→∞.

^13^
Three of these combinations, {+,+}, {−,−} and {+,−}, are subdivided when applied to real cases. Predation and parasitism, for example, share the {+,−} pair, but the kind of interaction and associated life cycles are different.

^14^
It is worth mentioning that there is a branch of theoretical ecology that deals with the *qualitative stability* of communities as derived from the sign matrix of pairwise interactions [288,289]. This indicates that much can be inferred solely based on the qualitative effects of member species on each other.

^15^
Importantly, thermodynamic arguments based on the maximum entropy formalism also reveal the power of constraints when dealing with the overall statistical patterns of ecosystem organization (instead of the internal logic) such as species–area relationships or abundance distributions in macroecology [294].

^16^
In this case, by making the summation over k we obtain $\Phi (𝐍)=\sum_{i,j,k}\alpha_{ij}^{k}N_{j}N_{i}$ as the associated dilution term.

^17^
Ignoring spatial effects, a mean-field approximation can be made for the average magnetization, which follows the differential equation $\frac{dM}{dt}=(T_{c}-T)M-M^{3}$. It is easy to show that three fixed points exist, associated with a symmetry-breaking (pitchfork) bifurcation [366]. A potential function (as defined in one-dimensional dynamical systems, see [258]) can be derived from dM/dt=−dV(M)/dM, i.e. from $V(M)=-\int \dot{M}\;dM$, which in this case, gives a fourth-order expression that is a symmetric function, i.e. $V(M)=-(T_{c}-T)M^{2}/2+M^{4}/4$. For $T>T_{c}$, the only stable state is M=0, whereas for $T<T_{c}$, the zero state is unstable, whereas two possible, completely symmetric solutions exist, namely $T_{\pm }=\pm \sqrt{T_{c}-T}$. These potential functions are displayed as insets in figure 10*a*.

^18^
Formally, this reduction can be described, in the thermodynamic limit, as indefinitely or infinitely lower [400,401].
